# Prognostic Significance of Isolated Beta 2-Microglobulin Elevation in Thai Multiple Myeloma: Impact of Renal Function Assessment

**DOI:** 10.14740/jh2212

**Published:** 2026-06-20

**Authors:** Pornpavee Siricharoenthai, Chanunya Thanoochan, Panachai Silpsamrit, Kannadit Prayongratana, Chonlada Laoruangroj, Rattapan Lamool, Tanapun Thamgrang

**Affiliations:** a Division of Hematology, Department of Medicine, Phramongkutklao Hospital, Bangkok, Thailand; b Division of Hematology, Department of Internal Medicine, Mahasarakham Hospital, Mahasarakham, Thailand

**Keywords:** Multiple myeloma, Prognosis, β_2_-microglobulin, Creatinine, Asian, Survival

## Abstract

**Background:**

The 2024 International Myeloma Society–International Myeloma Working Group consensus classifies isolated elevated β_2_-microglobulin (β2M) with normal serum creatinine (SCr) as high-risk multiple myeloma (MM). However, creatinine-based renal definitions may be inaccurate in Asian populations with lower muscle mass. We aimed to evaluate the prognostic impact of isolated elevated β2M on overall survival (OS) using SCr- and Cockcroft–Gault-derived creatinine clearance (CrCl)-based definitions.

**Methods:**

This retrospective study included newly diagnosed MM patients (2006–2023). Patients were classified as group A (β2M < 5.5 mg/L), group B (β2M ≥ 5.5 mg/L, SCr < 1.2 mg/dL), or group C (β2M ≥ 5.5 mg/L, SCr ≥ 1.2 mg/dL). In secondary analyses, renal function was redefined using CrCl (≥ 60 mL/min).

**Results:**

Among 250 patients (mean age 61.0 ± 12.1 years; 62.0% male), 34.4% underwent autologous stem cell transplantation. Groups A, B, and C included 109 (43.6%), 29 (11.6%), and 112 (44.8%) patients, respectively. At a median follow-up of 4.55 years, 4-year OS for groups A, B, and C was 79.8%, 71.6%, and 60.0% (P = 0.015). Group B did not demonstrate significantly inferior OS compared with group A (adjusted hazard ratio (aHR), 1.51; 95% confidence interval (CI), 0.70–3.24), whereas group C had significantly worse OS (aHR, 1.79; 95% CI, 1.11–2.86). When renal function was defined by CrCl, reclassified group B (n = 19) showed significantly inferior OS (aHR, 2.61; 95% CI, 1.17–5.86).

**Conclusions:**

Isolated β2M elevation showed a trend toward inferior OS using the SCr-based definition. CrCl-based reclassification appeared to improve prognostic stratification by identifying occult renal impairment not detected by SCr alone. These findings require validation in larger studies.

## Introduction

The International Staging System (ISS) and its subsequent revisions (R-ISS and R2-ISS) remain the cornerstone for global prognostic stratification in multiple myeloma (MM) [1]. These staging systems combine serum biomarkers, such as β_2_-microglobulin (β2M) and albumin, with cytogenetic risk factors to assign patients into distinct survival stages. However, interpretation of β2M levels within these frameworks is complicated by renal function, as impaired clearance can elevate β2M independently of tumor burden [2–4].

To address this limitation, the 2024 International Myeloma Society–International Myeloma Working Group (IMS–IMWG) consensus introduced a high-risk definition based on isolated β2M elevation (≥ 5.5 mg/L) in the context of preserved renal function (serum creatinine (SCr) < 1.2 mg/dL) [2, 5, 6]. This definition is intended to identify aggressive tumor biology independent of renal dysfunction and is supported by data from the CoMMpass [7, 8] and FORTE [9] studies, in which patients meeting this criterion demonstrated inferior survival comparable to genomically defined high-risk disease.

Although recent international guidelines have adopted revised staging systems and recognize isolated β2M elevation with normal creatinine as a high-risk feature [5, 10], its prognostic relevance in specific populations, including Thai patients, remains uncertain. Furthermore, the use of a fixed SCr threshold as a surrogate for preserved renal function may be problematic in real-world practice, as SCr is influenced by age, muscle mass, and ethnicity and may underestimate renal impairment, particularly in elderly and Asian populations [11–13].

We therefore evaluated the prognostic impact of the updated IMS–IMWG high-risk criterion—elevated β2M (≥ 5.5 mg/L) in the presence of preserved renal function—within a Thai cohort. Specifically, we compared survival outcomes among patients with isolated β2M elevation, standard-risk patients, and those with elevated β2M and renal impairment. In addition, we examined whether defining renal function using creatinine clearance (CrCl) provides improved prognostic stratification compared with a SCr-based definition for overall survival (OS) and progression-free survival (PFS).

## Materials and Methods

### Study design and population

This retrospective observational cohort study was conducted at Phramongkutklao Hospital, a tertiary military hospital in Thailand, between January 2006 and December 2023. We included consecutive patients with newly diagnosed MM, including both transplant-eligible and transplant-ineligible individuals, who received treatment at our institution during the study period.

### Objectives

The primary objective was to evaluate the independent prognostic value of isolated elevated serum β2M in patients with preserved renal function according to the 2024 IMS–IMWG high-risk criteria [2, 6], with comparison of OS across standard-risk patients, those with isolated β2M elevation, and those with renal impairment.

Secondary objectives included evaluation of PFS across these groups and assessment of OS and PFS using CrCl-based definitions of renal function.

Additional objectives included evaluation of treatment response to induction therapy, measured by objective response rate (ORR) and depth of response.

### Methods

All data were retrieved from the hospital electronic medical record system. Baseline variables included age, sex, Eastern Cooperative Oncology Group (ECOG) performance status, β2M, SCr, estimated CrCl, albumin, lactate dehydrogenase (LDH), hemoglobin (Hb), serum calcium, and high-risk cytogenetic abnormalities [14]. Treatment-related variables included induction regimen, best response to therapy, presence of plasmacytoma, and receipt of autologous stem cell transplantation (ASCT).

Patients were classified into three groups according to β2M level and renal function based on SCr: group A, standard risk (β2M < 5.5 mg/L, regardless of creatinine); group B, isolated β2M elevation (β2M ≥ 5.5 mg/L and creatinine < 1.2 mg/dL); and group C, β2M elevation with renal impairment (β2M ≥ 5.5 mg/L and creatinine ≥ 1.2 mg/dL).

For secondary analyses, renal function was redefined using CrCl calculated by the Cockcroft–Gault equation. A CrCl threshold of 60 mL/min was used to distinguish preserved renal function (≥ 60 mL/min) from renal impairment (< 60 mL/min). Under this classification, group B comprised patients with β2M ≥ 5.5 mg/L and CrCl ≥ 60 mL/min, and group C comprised those with β2M ≥ 5.5 mg/L and CrCl < 60 mL/min.

OS was defined as the time from diagnosis to death from any cause or last follow-up. PFS was defined as the time from diagnosis to disease progression, relapse, or death from any cause, whichever occurred first. Disease progression and relapse were assessed according to IMWG criteria [15].

### Determination of study size

The sample size was calculated based on the primary comparison between patients with isolated elevated β2M (group B) and standard-risk patients (group A). Based on survival outcomes reported in the FORTE dataset [2], a hazard ratio (HR) of 2.33 for group B was assumed, with a 70% survival probability in the control group. Given the low prevalence of isolated elevated β2M (approximately 5–6%) in the CoMMpass and FORTE datasets [2], an allocation ratio of 1:4 (group B to group A) was selected to ensure conservative power estimation. Using the Freedman method, a total of 45 events were required to achieve 80% power at a two-sided significance level of 0.05, corresponding to a minimum sample size of 26 patients in group B and 107 patients in group A. This calculation informed the decision to include the entire 17-year cohort to maximize statistical power.

### Statistical analysis

Categorical variables were summarized as frequencies and percentages, and continuous variables as mean ± standard deviation (SD) or median (interquartile range (IQR)), as appropriate. Baseline characteristics were compared across groups using the Chi-square or Fisher’s exact test for categorical variables and one-way analysis of variance (ANOVA) or Kruskal–Wallis test for continuous variables.

OS and PFS were estimated using the Kaplan–Meier method and compared using the log-rank test. Median follow-up was calculated using the reverse Kaplan–Meier method. Associations with OS and PFS were evaluated using Cox proportional hazards regression with the Efron method for ties. The proportional hazards assumption was assessed using Schoenfeld residuals.

Multivariable models included clinically relevant covariates known to be associated with outcomes (age, sex, proteasome inhibitor–based therapy, ASCT status, ECOG performance status, and plasmacytoma) [16–19], regardless of statistical significance in univariable analyses. High-risk cytogenetic abnormalities [17] were not included owing to substantial missing data.

All covariates in the primary multivariable models had complete data. LDH, despite its prognostic relevance [20], was excluded from the main models because of a high rate of missingness (38.8%). Sensitivity analyses were performed using multiple imputation by chained equations (50 datasets, predictive mean matching), incorporating all covariates, event indicators, and the Nelson–Aalen estimator; estimates were combined using Rubin’s rules.

An additional sensitivity analysis used a stratified Cox model by era of diagnosis to account for temporal changes in treatment and supportive care. Kaplan–Meier and Cox analyses were conducted using identical model specifications for both SCr- and CrCl-based classifications.

All analyses were performed using Stata version 17.0 (StataCorp LLC, College Station, TX, USA). Two-sided P values < 0.05 were considered statistically significant.

### Ethics approval

This study was approved by the Institutional Review Board of the Royal Thai Army Medical Department (Approval Number IRBRTA 1669/2566). The study was conducted in accordance with the principles of the Declaration of Helsinki and adhered to the International Conference on Harmonization Good Clinical Practice (ICH-GCP) guidelines.

## Results

### Baseline characteristics

A total of 250 patients diagnosed with MM during the study period were included. Of these, 109 patients (43.6%) were classified as group A (standard risk), 29 (11.6%) as group B (isolated elevated β2M), and 112 (44.8%) as group C (elevated β2M with renal impairment).

The mean age at diagnosis was 60.98 ± 12.08 years and did not differ significantly among the three groups. Overall, 155 patients (62.0%) were male; however, the proportion of male patients was significantly lower in group B (eight patients, 27.6%) compared with the other groups (P < 0.001). Most patients (54.4%) had an ECOG performance status of 0–1 at diagnosis.

The ISS classified 35 patients (14.0%) as stage I, 74 (29.6%) as stage II, and 141 (56.4%) as stage III. Cytogenetic data required for R-ISS risk stratification were unavailable in 195 patients (78.0%). Among the 55 patients (22.0%) with available cytogenetic information, high-risk abnormalities were identified in 13 patients, including t(4;14) in six patients (2.4%) and del(17p) in seven patients (2.8%).

A total of 86 patients (34.4%) in the cohort underwent ASCT, including 42 patients (38.5%) in group A, seven (24.1%) in group B, and 37 (33.0%) in group C. The distribution of ASCT did not differ significantly among the three groups (P = 0.346).

Additional baseline characteristics and laboratory parameters are summarized in Table 1.

**Table 1:** Baseline Characteristics

Parameter	Total, N = 250 (100%)	Standard risk; group A, N = 109 (43.6%)	Isolated high β2M; group B, N = 29 (11.6%)	High β2M with renal impairment; group C, N = 112 (44.8%)	P value
Year of diagnosis, no. (%)					
2006–2010	55 (22.00)	23 (21.10)	11 (37.93)	21 (18.75)	0.354
2011–2015	70 (28.00)	35 (31.82)	6 (20.69)	29 (26.13)	
2016–2020	102 (40.80)	41 (37.27)	11 (37.93)	50 (45.05)	
2021–2023	23 (9.20)	10 (9.09)	1 (3.45)	12 (10.81)	
Age, years, ± SD	60.98 ± 12.08	59.89 ± 13.06	62.07 ± 11.64	61.77 ± 11.17	0.451
Sex, no. (%)					
Male	155 (62.00)	70 (64.22)	8 (27.59)	77 (68.75)	< 0.001
Female	95 (38.00)	39 (35.78)	21 (72.41)	35 (31.25)	
Weight, kg, ± SD	58.88 ± 12.08	60.65 ± 12.32	55.78 ± 10.19	57.96 ± 12.11	0.085
ECOG, no. (%)					
0–1	136 (54.40)	71 (65.14)	15 (51.72)	50 (44.64)	0.049
2	74 (29.60)	26 (23.85)	7 (24.14)	41 (36.61)	
3	29 (11.60)	8 (7.34)	6 (20.69)	15 (13.39)	
4	11 (4.40)	4 (3.67)	1 (3.45)	6 (5.36)	
β_2_-microglobulin, mg/L (IQR)	5.99 (3.75–12.16)	3.47 (2.40–4.30)	7.17 (5.78–9)	12.64 (8.47–17.57)	< 0.001
Creatinine, mg/dL (IQR)	1.30 (0.90–2.32)	0.92 (0.71–1.10)	0.90 (0.73–0.97)	2.54 (1.57–5.40)	< 0.001
CrCl (Cockcroft-Gault Equation), mL/min (IQR)	47.07 (21.58–68.52)	62.12 (48.94–87.76)	61.62 (47.90–76.50)	19.53 (10.74–35.62)	< 0.001
Estimated GFR (CKD-EPI 2021), mL/min/1.73 m^2^ (IQR)	57.83 (28.24–86.67)	82.90 (65.34–100.30)	84.30 (68.77–96.93)	22.62 (9.41–46.61)	< 0.001
Albumin, mg/dL ± SD, n = 249	3.43 ± 0.78	3.56 ± 0.69	3.27 ± 0.69	3.33 ± 0.86	0.050
High LDH, no. (%), n = 153	58 (37.91)	17 (26.15)	11 (64.71)	30 (42.25)	0.009
High-risk cytogenetics, no. (%)					
No detection	42 (16.80)	19 (17.43)	7 (24.14)	16 (14.29)	0.457
t(4:14)	6 (2.40)	2 (1.83)	0 (0.00)	4 (3.57)	
Del(17p)	7 (2.80)	1 (0.92)	1 (3.45)	5 (4.46)	
Not done	195 (78.00)	87 (79.82)	21 (72.41)	87 (77.68)	
International Staging System (ISS), no. (%)					
Stage I	35 (14.00)	35 (32.11)	0 (0.00)	0 (0.00)	< 0.001
Stage II	74 (29.60)	74 (67.89)	0 (0.00)	0 (0.00)	
Stage III	141 (56.40)	0 (0.00)	29 (100.00)	112 (100.00)	
Hemoglobin, g/dL (IQR)	8.9 (7.5–10.2)	9.6 (8–11)	9.2 (8–9.7)	8.2 (6.9–9.4)	< 0.001
Calcium, mg/dL (IQR)	9.54 (9–10.69)	9.37 (8.91–9.75)	9.25 (8.8–9.6)	10.1 (9.13–11.9)	< 0.001
Induction regimen, no. (%)					
Bortezomib-based	126 (50.40)	52 (47.71)	8 (27.59)	66 (58.59)	0.008
Thalidomide-based	72 (28.80)	30 (27.52)	12 (41.38)	30 (26.79)	0.282
Lenalidomide-based	5 (2.00)	4 (3.67)	0 (0.00)	1 (0.89)	0.260
Daratumumab-based	1 (0.40)	0 (0.00)	0 (0.00)	1 (0.89)	1.000
Type of induction regimen, no. (%)					
Palliative care/steroid monotherapy	15 (6.00)	7 (6.42)	2 (6.90)	6 (5.36)	0.122
Doublet	84 (33.60)	31 (28.44)	16 (55.17)	37 (33.04)	
Triplet	130 (52.00)	58 (53.21)	10 (34.48)	62 (55.36)	
Quadruplet	1 (0.40)	0 (0.00)	0 (0.00)	1(0.89)	
Chemotherapy	20 (8.00)	13 (11.93)	1 (3.45)	6 (5.36)	
Best response after induction, no. (%), n = 218					
sCR	31 (14.22)	12 (12.24)	2 (8.33)	17 (17.71)	0.388
CR	51 (23.39)	26 (26.53)	7 (29.17)	18 (18.75)	
VGPR	47 (21.56)	23 (23.47)	1 (4.17)	23 (23.96)	
PR	57 (26.15)	25 (25.51)	10 (41.67)	22 (22.92)	
SD	25 (11.47)	9 (9.18)	3 (12.50)	13 (13.54)	
PD	7 (3.21)	3 (3.06)	1 (4.17)	3 (3.13)	
ORR^a^, no. (%), n = 218	186 (85.32)	86 (87.76)	20 (83.33)	80 (83.33)	0.630
Plasmacytoma^b^, no. (%)	83 (33.20)	41 (37.61)	12 (41.38)	30 (26.79)	0.141
ASCT, no. (%)	86 (34.40)	42 (38.53)	7 (24.14)	37 (33.04)	0.346
Mortality, no. (%)	89 (35.60)	32 (29.36)	11 (37.93)	46 (41.07)	0.192

^a^Defined as a response of PR or better. ^b^No specified sites and numbers. SD: standard deviation; IQR: interquartile range; CrCl: creatinine clearance; GFR: glomerular filtration rate; β2M: β_2_-microglobulin; sCR: stringent complete response; CR: complete response; VGPR: very good partial response; ORR: objective response rate; PR: partial response; SD: stable disease; PD: progressive disease; ECOG: Eastern Cooperative Oncology Group.

### Induction regimens and responses

Among novel agent-based first-line induction therapies, bortezomib-based regimens were the most frequently used, accounting for 50.4% of all patients. The use of bortezomib-based induction was significantly less common in group B (27.6%) than in group A (47.7%) and group C (58.6%) (P = 0.008). Thalidomide was the second most commonly prescribed novel agent, used in 28.8% of patients. Due to reimbursement constraints and cost considerations, lenalidomide-based induction therapy was infrequently administered (2.0%), while daratumumab-based induction was used in only one patient (0.4%). Triplet-based induction regimens were the most commonly used induction approach, administered to 52.0% of patients.

**Figure 1:**
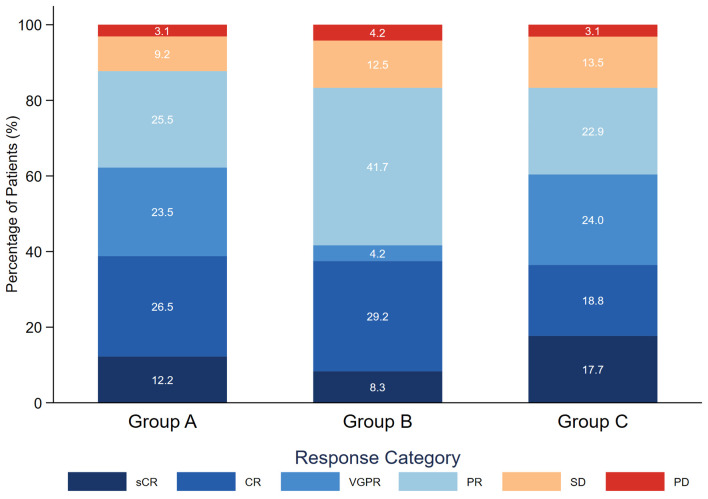
Best treatment response following induction therapy. Stacked bar chart illustrating the distribution of the best response levels among patients with newly diagnosed multiple myeloma, stratified by prognostic group: group A (standard risk: β2M < 5.5 mg/L), group B (isolated high β2M: β2M ≥ 5.5 mg/L with creatinine < 1.2 mg/dL), and group C (high β2M with renal impairment: β2M ≥ 5.5 mg/L with creatinine ≥ 1.2 mg/dL). Percentages within each bar represent the proportion of patients achieving each response category. Response categories are defined according to IMWG criteria, ranging from sCR to PD. Response data were available for 218 patients. β2M: β_2_-microglobulin; sCR: stringent complete response; CR: complete response; VGPR: very good partial response; PR: partial response; SD: stable disease; PD: progressive disease.

Response data were available for 218 patients. The ORR, defined as achieving at least a partial response, was 85.3% (186/218) and did not differ significantly among the three groups (P = 0.630). Likewise, the depth of best response after induction therapy was comparable across groups, with no significant differences observed (P = 0.388; Fig. 1).

### Overall survival

At a median follow-up of 4.55 years (95% confidence interval (CI), 4.14–5.22), 89 patients (35.6%) had died, including 32 patients (29.4%) in group A, 11 patients (37.9%) in group B, and 46 patients (41.1%) in group C.

The 4-year OS rates for groups A, B, and C were 79.8% (95% CI, 70.3–86.5), 71.6% (95% CI, 49.2–85.4), and 60.0% (95% CI, 48.7–69.6), respectively (P = 0.015).

**Figure 2:**
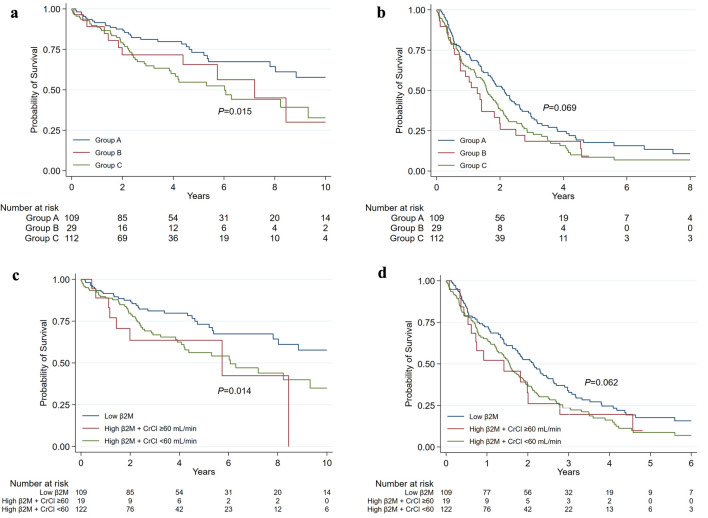
Survival outcomes stratified by β2M using serum creatinine– and creatinine clearance–based definitions of renal function. Kaplan–Meier estimates of overall survival (OS) and progression-free survival (PFS) among patients with newly diagnosed multiple myeloma. Panels (a) and (b) show OS and PFS, respectively, stratified by the 2024 IMS–IMWG serum creatinine–based criteria: group A (standard risk: β2M < 5.5 mg/L), group B (isolated high β2M: β2M ≥ 5.5 mg/L with serum creatinine < 1.2 mg/dL), and group C (high β2M with renal impairment: β2M ≥ 5.5 mg/L with serum creatinine ≥ 1.2 mg/dL). Panels (c) and (d) show OS and PFS, respectively, stratified by the creatinine clearance–based definition of renal function: low β2M, high β2M with preserved renal function (CrCl ≥ 60 mL/min), and high β2M with renal impairment (CrCl < 60 mL/min). IMS–IMWG: International Myeloma Society–International Myeloma Working Group; CrCl: creatinine clearance; β2M: β_2_-microglobulin.

The median OS for the overall cohort was 8.44 years (95% CI, 6.07–13.67). Median OS was not reached (NR) in group A, whereas it was 7.21 years (95% CI, 1.99–NR) in group B and 6.03 years (95% CI, 3.91–9.32) in group C (Fig. 2a).

In univariable Cox analysis, group B demonstrated a higher risk of death compared with group A (HR, 1.64; 95% CI, 0.82–3.26), although this did not reach statistical significance, whereas group C had significantly inferior OS. No significant difference in OS was observed between groups B and C. Multivariable analysis showed a consistent pattern, with group B demonstrating a nonsignificant increase in mortality compared with group A (adjusted hazard ratio (aHR), 1.51; 95% CI, 0.70–3.24), whereas group C was independently associated with inferior OS (Table 2; Supplementary Material 1, jh.elmerpub.com).

**Table 2:** Univariable and Multivariable Cox Regression Analyses for Overall Survival and Progression-Free Survival Stratified by β2M and SCr

Prognostic group	Overall survival				Progression-free survival			
	Crude HR (95% CI)	P	Adjusted HR (95% CI)^a^	P	Crude HR (95% CI)	P	Adjusted HR (95% CI)^a^	P
Group A (standard)	1.00 (Ref)	-	1.00 (Ref)	-	1.00 (Ref)	-	1.00 (Ref)	-
Group B (isolated high β2M)	1.64 (0.82–3.26)	0.159	1.51 (0.70–3.24)	0.291	1.53 (0.98–2.39)	0.064	1.37 (0.86–2.20)	0.187
Group C (high β2M and impaired renal function)	1.96 (1.24–3.09)	0.004	1.79 (1.11–2.86)	0.016	1.38 (1.03–1.85)	0.033	1.31 (0.96–1.79)	0.091
Comparison group B vs. C	0.84 (0.43–1.62)	0.596	0.85 (0.40–1.81)	0.667	1.11 (0.71–1.73)	0.647	1.05 (0.64–1.71)	0.851

^a^Adjusted for age, sex, bortezomib-based regimens, ASCT status, Eastern Cooperative Oncology Group performance status (≥ 2), and presence of plasmacytoma. HR: hazard ratio; CI: confidence interval; β2M: β_2_-microglobulin; SCr: serum creatinine; ASCT: autologous stem cell transplantation.

### Progression-free survival

The 4-year PFS rates for groups A, B, and C were 24.6% (95% CI, 16.5–33.5), 18.4% (95% CI, 6.8–34.6), and 15.7% (95% CI, 9.0–24.2), respectively (P = 0.069).

The median PFS for the overall cohort was 1.75 years (95% CI, 1.45–1.99). Median PFS was 2.13 years (95% CI, 1.63–2.62) in group A, 1.28 years (95% CI, 0.73–1.99) in group B, and 1.59 years (95% CI, 1.23–1.94) in group C (Fig. 2b).

In univariable analysis, group B showed a trend toward inferior PFS compared with group A that did not reach statistical significance (HR, 1.53; 95% CI, 0.98–2.39). This association was attenuated in multivariable analysis (aHR, 1.37; 95% CI, 0.86–2.20). No statistically significant differences in PFS were observed between groups B and C (Table 2; Supplementary Material 1, jh.elmerpub.com).

### Survival outcomes by β2M and CrCl

**Figure 3:**
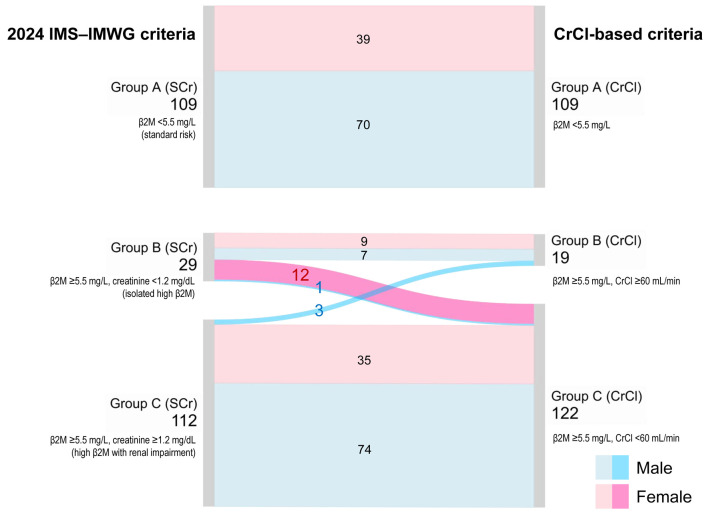
Reclassification of prognostic groups according to the 2024 IMS–IMWG criteria and the CrCl-based criteria. The Sankey diagram illustrates changes in patient distribution when transitioning from the standard SCr-based definition (left) to a CrCl-based threshold of 60 mL/min (right). Flow colors represent sex (pink for female and blue for male), while nodes indicate prognostic groups defined by β2M and renal function. IMS–IMWG: International Myeloma Society–International Myeloma Working Group; SCr: serum creatinine; CrCl: creatinine clearance; β2M: β_2_-microglobulin.

After reclassifying patients in groups B and C using CrCl instead of SCr, with a cutoff of 60 mL/min, 19 patients (7.6%) were reclassified into group B, whereas 122 patients (48.8%) were reclassified into group C. The resulting changes in patient distribution are illustrated in Figure 3.

For OS, in univariable analyses, both groups B and C demonstrated inferior survival compared with group A, and these associations remained statistically significant after multivariable adjustment. Compared with group A, group B had an aHR for death of 2.61 (95% CI, 1.17–5.86). No statistically significant difference in OS was observed between groups B and C, consistent with the SCr-based stratification (Table 3; Fig. 2c).

**Table 3:** Univariable and Multivariable Cox Regression Analyses for Overall Survival and Progression-Free Survival Stratified by β2M and CrCl

Prognostic group	Overall survival				Progression-free survival			
	Crude HR (95% CI)	P	Adjusted HR (95% CI)^a^	P	Crude HR (95% CI)	P	Adjusted HR (95% CI)^a^	P
Group A (standard)	1.00 (Ref)	-	1.00 (Ref)	-	1.00 (Ref)	-	1.00 (Ref)	-
Group B (isolated high β2M)	2.23 (1.02–4.88)	0.044	2.61 (1.17–5.86)	0.020	1.47 (0.85–2.55)	0.172	1.39 (0.80–2.44)	0.243
Group C (high β2M and impaired renal function)	1.84 (1.17– 2.88)	0.008	1.63 (1.02–2.59)	0.040	1.40 (1.05–1.86)	0.023	1.31 (0.97–1.79)	0.078
Comparison group B vs. C	1.21 (0.57–2.57)	0.612	1.61 (0.73–3.53)	0.239	1.05 (0.61– 1.81)	0.857	1.06 (0.60–1.86)	0.835

^a^Adjusted for age, sex, bortezomib-based regimens, ASCT status, Eastern Cooperative Oncology Group performance status (≥ 2), and presence of plasmacytoma. HR: hazard ratio; CI: confidence interval; β2M: β_2_-microglobulin; CrCl: creatinine clearance; ASCT: autologous stem cell transplantation.

For PFS, univariable analyses showed a nonsignificant trend toward inferior PFS in group B compared with group A, whereas group C had significantly worse PFS. No significant difference in PFS was observed between groups B and C, consistent with the SCr-based stratification. In multivariable analyses, these associations were attenuated and no longer statistically significant for either group (Table 3; Fig. 2d; Supplementary Material 2, jh.elmerpub.com).

Notably, among patients who underwent ASCT (n = 86), the association between isolated high β2M and OS appeared stronger when renal function was defined using CrCl rather than SCr; however, this association did not reach conventional statistical significance (HR, 3.11 (CrCl); 95% CI 0.62–15.53 versus HR, 1.69 (SCr); 95% CI 0.82–3.26) (Supplementary Material 3, jh.elmerpub.com).

### Sensitivity analyses

In sensitivity analyses incorporating imputed LDH values, the aHRs for OS were consistent across groups defined by SCr (aHR, 1.42; 95% CI, 0.65–3.10) and by CrCl (aHR, 2.53; 95% CI, 1.12–5.69) (Supplementary Material 4, jh.elmerpub.com).

In an additional sensitivity analysis stratified by era of diagnosis, the CrCl-based definition remained prognostically significant, with group B showing a higher risk of death than group A (aHR, 2.67; 95% CI, 1.17–6.07), consistent with the primary analysis (Supplementary Materials 5 and 6, jh.elmerpub.com).

## Discussion

This study highlights the prognostic implications of the updated IMS–IMWG consensus [2, 6], which defines elevated serum β2M (≥ 5.5 mg/L) in the presence of preserved renal function (SCr < 1.2 mg/dL) as a high-risk feature in MM. Patients meeting this criterion (group B) exhibited a consistent trend toward inferior outcomes compared with the standard-risk group. Although statistical significance was not observed for all endpoints, the effect estimates were directionally consistent with an increased risk of progression and death. In a secondary exploratory analysis using CrCl-based reclassification, the size of this subgroup was reduced, and a statistically significant association with inferior OS was observed in the isolated β2M group.

The IMS–IMWG definition relies on SCr, which may underestimate renal dysfunction in elderly individuals and those with low muscle mass, particularly in Asian populations [11–13]. In our cohort, SCr-based classification demonstrated limited prognostic stratification, whereas CrCl-based reclassification identified a subgroup with a stronger association with adverse survival outcomes. These findings suggest that reliance on SCr alone may have limitations in risk stratification among high-risk patients when renal function is assessed using this marker.

Notably, the sex distribution differed across risk groups when renal function was defined using SCr, with fewer males in group B. Reclassification using CrCl resulted in substantial redistribution, with many patients previously categorized as having isolated β2M elevation being reassigned as having occult renal impairment, particularly among females (12 of 21 female patients, 57.1%). This finding underscores the clinical utility of CrCl as a simple and practical measure for detecting occult renal impairment in MM, particularly among female patients in whom SCr alone may underestimate renal dysfunction. Accordingly, sex was included as a covariate in all multivariable models to account for potential confounding.

The HR for CrCl-reclassified group B in our analysis was consistent with findings from the CoMMpass [7, 8] and FORTE [9] studies, in which isolated β2M elevation was associated with inferior OS compared with standard-risk disease (CoMMpass: HR, 1.79; 95% CI, 1.09–2.94; P = 0.02; FORTE: HR, 2.33; 95% CI, 0.88–6.25; P = 0.091). The concordance in effect sizes across these studies suggests that CrCl-based reclassification may help identify patients with occult renal impairment who are not fully captured by fixed SCr thresholds. Notably, wide CIs were also observed in the CoMMpass and FORTE studies, likely reflecting the relatively small number of patients within this subgroup in both real-world and clinical trial populations.

Although the CoMMpass and FORTE trials included only transplant-eligible patients, our subgroup analysis of patients who underwent ASCT demonstrated a similar association between isolated elevated β2M and OS when renal function was defined using CrCl. Notably, the magnitude of the effect was greater in this subgroup. However, the wide CI, reflecting the limited sample size, warrants cautious interpretation. Accordingly, the primary interpretation should rely on the overall cohort, while subgroup and sensitivity analyses are intended to support the robustness of the findings rather than serve as definitive estimates of effect.

Inferior outcomes in group B may also partly reflect treatment disparities, particularly lower exposure to bortezomib-based therapy. During the study period, access to bortezomib in Thailand was restricted by reimbursement policies, which may have resulted in undertreatment of patients with high-risk disease but normal SCr. Although bortezomib-based induction was included as a covariate in multivariable models, residual confounding cannot be excluded. This residual treatment imbalance should therefore be considered when interpreting the independent prognostic effect of isolated β2M elevation in group B.

The absence of significant differences in PFS, despite stronger associations with OS, is notable. This pattern suggests that while progression occurred across risk groups, high-risk patients were more likely to die after relapse, underscoring the importance of post-relapse management. Given the susceptibility of retrospective PFS assessment to bias from non-standardized imaging and follow-up, OS represents a more robust endpoint in this study.

The primary limitations of this study include substantial missing data for cytogenetic testing (78.0%) and LDH levels (38.8%). Owing to the real-world setting in a low- to middle-income country, comprehensive cytogenetic risk profiling was not routinely performed for all patients, despite its central role in the R-ISS, R2-ISS, and the updated IMS–IMWG high-risk classification. Consequently, misclassification of risk groups cannot be excluded. Systematic bias is also possible, as patients with more severe disease or those treated in earlier periods may have been less likely to undergo cytogenetic testing. However, because cytogenetic testing was not reimbursed during the study period, temporal (era-related) differences were unlikely to be the primary driver of the missing data.

Given the small number of patients with documented high-risk cytogenetic abnormalities, these individuals were not excluded from the analysis. Consequently, group A may not represent a purely standard-risk population. However, excluding such patients—particularly in the setting of extensive missing cytogenetic data—would likely have introduced greater selection bias, a well-recognized limitation of retrospective studies. Conversely, group B may have harbored a higher burden of unmeasured high-risk cytogenetic abnormalities, representing a potential source of unmeasured confounding that could have limited accurate risk prognostication. Taken together, this high proportion of missing cytogenetic data may have introduced substantial residual confounding, which should be considered when interpreting the independent prognostic value of isolated β2M elevation in our cohort.

This study included a cohort of patients with MM followed over a 17-year period. The prolonged study duration likely introduced heterogeneity in treatment availability and supportive care. Although era-stratified sensitivity analyses were performed, residual confounding may persist. In addition, both bortezomib exposure and ASCT were included as covariates to partially account for treatment heterogeneity. Other evolving treatment components—including immunomodulatory drugs, maintenance strategies, and salvage therapies—were not fully captured due to the limited number of outcome events and incomplete longitudinal treatment data.

The inclusion of both transplant-eligible and transplant-ineligible patients may complicate interpretation. ASCT was therefore included as a time-fixed binary covariate in the multivariable model because precise transplant dates were unavailable. Although this approach may introduce immortal time bias, ASCT was retained to account for the substantial survival benefit associated with treatment intensification. Accordingly, the HR for ASCT should be interpreted with caution, as its inclusion was intended to adjust for treatment heterogeneity rather than to estimate the effect of transplantation itself.

Another limitation is the small number of patients in group B (n = 29). Although this met the prespecified sample size requirement, the limited statistical power renders the lack of a statistically significant difference in OS between groups B and A inconclusive, rather than indicative of true equivalence or a negative finding. Crucially, the observed point estimates in our cohort consistently support the adverse prognostic directionality of the 2024 IMS–IMWG high-risk criterion. Furthermore, after exploratory reclassification using CrCl, the number of patients in group B decreased further (n = 19), at which point a statistically significant association with inferior OS became apparent. Rather than validating an ethnicity-specific creatinine threshold, this finding suggests that CrCl-based stratification primarily optimizes risk assessment by capturing occult renal impairment that is otherwise masked by a fixed SCr threshold. Nonetheless, given the limited sample size in this subgroup, these results warrant cautious interpretation and should be viewed as hypothesis-generating.

Although CrCl may better reflect glomerular filtration rate (GFR) than SCr alone, the Cockcroft–Gault equation has important limitations, particularly in elderly individuals and those with obesity or low muscle mass. Newer equations, such as the 2021 Chronic Kidney Disease Epidemiology Collaboration (CKD-EPI) equation, may provide more accurate estimates of GFR [21]. However, because drug dosing recommendations and diagnostic criteria for MM still rely on Cockcroft–Gault–derived CrCl, this measure was used [22–24].

This study was conducted at a single military hospital in Thailand, which may limit generalizability. In addition, evolving treatment paradigms incorporating triplet and quadruplet regimens [5] may attenuate the prognostic impact of individual biomarkers such as β2M. Prospective studies in diverse populations treated with contemporary regimens are therefore needed to validate these findings and to directly compare SCr–, Cockcroft–Gault–derived CrCl, and alternative equation-based renal function measures, including CKD-EPI–based estimated GFR, for risk stratification and prognostic discrimination.

The lack of a statistically significant survival difference between group B and group C limits our ability to conclude that these groups represent distinct biological phenotypes. The primary clinical utility of incorporating CrCl in this context is therefore not to define a novel biological subtype, but rather to optimize risk stratification by reducing the potential misclassification of patients with occult renal impairment into the standard-risk category.

Importantly, our findings highlight a pragmatic, low-cost approach to risk stratification. By using elevated β2M in the context of preserved renal function as an independent prognostic marker, we provide a robust surrogate for identifying high-risk disease. This is especially critical in the absence of cytogenetic data, offering significant clinical value in resource-limited settings worldwide.

In conclusion, our findings generally support the prognostic relevance of the 2024 IMS–IMWG definition of isolated β2M elevation. However, statistical precision was limited by the small number of patients in group B and the potential underestimation of renal impairment by SCr alone. Exploratory reclassification using CrCl appeared to improve prognostic stratification, likely by identifying occult renal dysfunction not captured by SCr. Nevertheless, these findings should be considered hypothesis-generating and warrant validation in larger contemporary cohorts.

## Supplementary Material

Suppl 1 Multivariable Cox proportional hazards analysis for overall survival (OS) and progression-free survival (PFS) stratified by β_2_-microglobulin (β2M) and serum creatinine.

Suppl 2 Multivariable Cox proportional hazards analysis for overall survival (OS) and progression-free survival (PFS) stratified by β_2_-microglobulin (β2M) and creatinine clearance (CrCl).

Suppl 3 Kaplan–Meier estimates of overall survival (OS) in patients with newly diagnosed multiple myeloma undergoing ASCT, stratified by β2-microglobulin (β2M) levels and renal function using two different definitions.

Suppl 4 Sensitivity analysis of overall survival (OS) using a multivariable Cox proportional hazards model after multiple imputation of missing LDH and albumin values by chained equations (MICE).

Suppl 5 Stratified multivariable Cox proportional hazards analysis for overall survival (OS). The model was stratified by era of diagnosis (2006–2010, 2011–2015, 2016–2020, and 2021–2023) to account for potential variation in baseline hazards across treatment eras.

Suppl 6 Stratified multivariable Cox proportional hazards analysis for overall survival (OS). The model was stratified by era of diagnosis (2006–2010, 2011–2015, 2016–2020, and 2021–2023) to account for potential variation in baseline hazards across treatment eras.

## Data Availability

The datasets generated during and/or analyzed during the current study are available from the corresponding author on reasonable request.

## Abbreviations

aHR: adjusted hazard ratio
ASCT: autologous stem cell transplantation
ANOVA: analysis of variance
β2M: β_2_-microglobulin
CI: confidence interval
CKD-EPI: Chronic Kidney Disease Epidemiology Collaboration
CrCl: creatinine clearance
ECOG: Eastern Cooperative Oncology Group
GFR: glomerular filtration rate
Hb: hemoglobin
HR: hazard ratio
ICH-GCP: International Conference on Harmonization Good Clinical Practice
IMS–IMWG: International Myeloma Society–International Myeloma Working Group
IMWG: International Myeloma Working Group
IQR: interquartile range
IRB: Institutional Review Board
ISS: International Staging System
LDH: lactate dehydrogenase
MM: multiple myeloma
NR: not reached
ORR: objective response rate
OS: overall survival
PFS: progression-free survival
R-ISS: Revised International Staging System
R2-ISS: Second Revision of the International Staging System
SCr: serum creatinine
SD: standard deviation
TSH: Thai Society of Hematology
