# Supplementary material for: Prognostic Significance of Isolated Beta 2-Microglobulin Elevation in Thai Multiple Myeloma: Impact of Renal Function Assessment
Source: J Hematol. 2026 Jun 20;15(3):144–55. doi: 10.14740/jh2212 (PMC13375423; doi:10.14740/jh2212)
Supplement: Suppl 1 — Multivariable Cox proportional hazards analysis for overall survival (OS) and progression-free survival (PFS) stratified by β2-microglobulin (β2M) and serum creatinine. [file jh-15-03-144-s001.docx]

**Suppl 1.** Multivariable Cox proportional hazards analysis for overall survival (OS) and progression-free survival (PFS) stratified by β_2_-microglobulin (β2M) and serum creatinine.

| **Variable** | **Overall Survival (OS)** | | **Progression-Free Survival (PFS)** | |
| --- | --- | --- | --- | --- |
|  | **aHR (95% CI)** | ***P* value** | **aHR (95% CI)** | ***P* value** |
| Study Groups |  |  |  |  |
| Group A (Standard) | Reference | — | Reference | — |
| Group B (Isolated high β2M) | 1.51 (0.70–3.24) | 0.291 | 1.37 (0.86–2.20) | 0.187 |
| Group C (High β2M and impaired renal function) | 1.79 (1.11–2.86) | 0.016 | 1.31 (0.96–1.79) | 0.091 |
| Patient Characteristics |  |  |  |  |
| Age (per year increase) | 1.00 (0.98–1.03) | 0.670 | 0.99 (0.98–1.01) | 0.617 |
| Gender (Male vs. Female) | 1.74 (1.02–2.95) | 0.040 | 1.09 (0.80–1.48) | 0.581 |
| ECOG performance status (≥2 vs. 0-1) | 2.19 (1.36–3.54) | 0.001 | 1.62 (1.20–2.20) | 0.002 |
| Disease & Treatment |  |  |  |  |
| Plasmacytoma (Yes vs. No) | 1.29 (0.83–2.02) | 0.261 | 1.13 (0.84–1.54) | 0.420 |
| ASCT (Yes vs. No) | 0.43 (0.23–0.81) | 0.009 | 0.48 (0.32–0.72) | <0.001 |
| Bortezomib induction | 0.72 (0.46–1.11) | 0.140 | 0.78 (0.58–1.03) | 0.084 |

*Abbreviations: aHR, adjusted hazard ratio; CI, confidence interval; β2M, β_2_-microglobulin; ASCT, autologous stem cell transplantation.*
