# Supplementary material for: Prognostic Significance of Isolated Beta 2-Microglobulin Elevation in Thai Multiple Myeloma: Impact of Renal Function Assessment
Source: J Hematol. 2026 Jun 20;15(3):144–55. doi: 10.14740/jh2212 (PMC13375423; doi:10.14740/jh2212)
Supplement: Suppl 2 — Multivariable Cox proportional hazards analysis for overall survival (OS) and progression-free survival (PFS) stratified by β2-microglobulin (β2M) and creatinine clearance (CrCl). [file jh-15-03-144-s002.docx]

**Suppl 2.** Multivariable Cox proportional hazards analysis for overall survival (OS) and progression-free survival (PFS) stratified by β_2_-microglobulin (β2M) and creatinine clearance (CrCl).

| **Variable** | **Overall Survival (OS)** | | **Progression-Free Survival (PFS)** | |
| --- | --- | --- | --- | --- |
|  | **aHR (95% CI)** | ***P* value** | **aHR (95% CI)** | ***P* value** |
| Study Groups |  |  |  |  |
| Group A (Low β2M) | Reference | — | Reference | — |
| Group B (High β2M, CrCl ≥60 mL/min) | 2.61 (1.17–5.86) | 0.020 | 1.39 (0.80–2.44) | 0.243 |
| Group C (High β2M, CrCl <60 mL/min) | 1.63 (1.02–2.59) | 0.040 | 1.31 (0.97–1.78) | 0.078 |
| Patient Characteristics |  |  |  |  |
| Age (per year) | 1.01 (0.98–1.03) | 0.596 | 1.00 (0.98–1.01) | 0.641 |
| Gender (Male vs. Female) | 1.90 (1.18–3.07) | 0.009 | 1.08 (0.81–1.44) | 0.596 |
| ECOG performance status (≥2 vs. 0–1) | 2.30 (1.42–3.72) | 0.001 | 1.62 (1.20–2.19) | 0.002 |
| Disease & Treatment |  |  |  |  |
| Plasmacytoma (Yes vs. No) | 1.29 (0.82–2.01) | 0.269 | 1.14 (0.84–1.54) | 0.411 |
| ASCT (Yes vs. No) | 0.45 (0.24–0.84) | 0.013 | 0.48 (0.32–0.72) | <0.001 |
| Bortezomib induction | 0.76 (0.49–1.17) | 0.209 | 0.78 (0.59–1.03) | 0.082 |

*Abbreviations: aHR, adjusted hazard ratio; CI, confidence interval; β2M, β_2_-microglobulin; ASCT, autologous stem cell transplantation.*
