# Supplementary material for: Prognostic Significance of Isolated Beta 2-Microglobulin Elevation in Thai Multiple Myeloma: Impact of Renal Function Assessment
Source: J Hematol. 2026 Jun 20;15(3):144–55. doi: 10.14740/jh2212 (PMC13375423; doi:10.14740/jh2212)
Supplement: Suppl 3 — Kaplan–Meier estimates of overall survival (OS) in patients with newly diagnosed multiple myeloma undergoing ASCT, stratified by β2-microglobulin (β2M) levels and renal function using two different definitions. [file jh-15-03-144-s003.docx]

**Suppl 3.** Kaplan–Meier estimates of overall survival (OS) in patients with newly diagnosed multiple myeloma undergoing ASCT, stratified by β2-microglobulin (β2M) levels and renal function using two different definitions.

**(A)** Stratification based on serum creatinine: Group A (standard risk: β2M <5.5 mg/L), Group B (isolated high β2M: β2M ≥5.5 mg/L with creatinine <1.2 mg/dL), and Group C (high β2M with renal impairment: β2M ≥5.5 mg/L with creatinine ≥1.2 mg/dL).

| **Prognostic Group** | **Unadjusted HR (95% CI)** | ***P* value** |
| --- | --- | --- |
| **Group A** (Standard) | 1.00 (Ref) | - |
| **Group B** (Isolated high β2M) | 1.69 (0.82-3.26) | 0.159 |
| **Group C** (High β2M and impaired renal function) | 1.95 (1.24-3.09) | 0.004 |
|  |  |  |
| **Comparison Group B vs. C** | 0.84 (0.43-1.62) | 0.596 |

**(B)** Stratification based on creatinine clearance (CrCl): low β2M (<5.5 mg/L), high β2M (≥5.5 mg/L) with preserved renal function (CrCl ≥60 mL/min), and high β2M (≥5.5 mg/L) with renal impairment (CrCl <60 mL/min).

| **Prognostic Group** | **Unadjusted HR (95% CI)** | ***P* value** |
| --- | --- | --- |
| **Group A:** low β2M (≤5.5 mg/L) | 1.00 (Ref) | - |
| **Group B:** high β2M (> 5.5 mg/L) with preserved renal function (CrCl ≥60 mL/min) | 3.11 (0.62-15.53) | 0.166 |
| **Group C:** high β2M (>5.5 mg/L) with renal impairment (CrCl <60 mL/min) | 2.21 (0.82-5.92) | 0.117 |
|  |  |  |
| **Comparison Group B vs. C** | 1.41 (0.31-6.50) | 0.658 |

*Abbreviations: HR, hazard ratio; CI, confidence interval; β2M, β_2_-microglobulin; ASCT, autologous stem cell transplantation*
