# Supplementary material for: Prognostic Significance of Isolated Beta 2-Microglobulin Elevation in Thai Multiple Myeloma: Impact of Renal Function Assessment
Source: J Hematol. 2026 Jun 20;15(3):144–55. doi: 10.14740/jh2212 (PMC13375423; doi:10.14740/jh2212)
Supplement: Suppl 4 — Sensitivity analysis of overall survival (OS) using a multivariable Cox proportional hazards model after multiple imputation of missing LDH and albumin values by chained equations (MICE). [file jh-15-03-144-s004.docx]

**Suppl 4.** Sensitivity analysis of overall survival (OS) using a multivariable Cox proportional hazards model after multiple imputation of missing LDH and albumin values by chained equations (MICE).

| **Variable** | **Model 1: Standard Criteria (Creatinine)** | | **Model 2: Modified Criteria (CrCl)** | |
| --- | --- | --- | --- | --- |
|  | Adjusted HR (95% CI)* | *P* value | Adjusted HR (95% CI)* | *P* value |
| **Risk Group (Ref: Group A)** |  |  |  |  |
| Group B (Isolated high β2M) | 1.42 (0.65–3.10) | 0.376 | 2.53 (1.12–5.69) | 0.025 |
| Group C (High β2M + impaired renal function) | 1.79 (1.11–2.88) | 0.016 | 1.62 (1.01–2.58) | 0.044 |
|  |  |  |  |  |
| **Covariates** |  |  |  |  |
| Age (per year) | 1.01 (0.98–1.03) | 0.563 | 1.01 (0.98–1.03) | 0.510 |
| Male gender | 1.69 (0.99–2.89) | 0.053 | 1.89 (1.17–3.06) | 0.010 |
| Bortezomib induction | 0.73 (0.47–1.13) | 0.159 | 0.77 (0.49–1.19) | 0.237 |
| Autologous SCT | 0.43 (0.23–0.82) | 0.011 | 0.45 (0.24–0.86) | 0.015 |
| Plasmacytoma | 1.27 (0.80–2.03) | 0.315 | 1.26 (0.79–2.02) | 0.333 |
| ECOG performance status ≥2 | 2.07 (1.27–3.39) | 0.004 | 2.19 (1.34–3.57) | 0.002 |
| LDH (per U/L) | 1.00 (1.00–1.00) | 0.513 | 1.00 (1.00–1.00) | 0.532 |
| Albumin <3.5 mg/L | 1.22 (0.79–1.90) | 0.373 | 1.20 (0.77–1.86) | 0.417 |

*Adjusted for age, sex, bortezomib-based regimens, ASCT status, Eastern Cooperative Oncology Group performance status (≥2), LDH (imputed), low albumin (imputed), and presence of plasmacytoma

*Abbreviations: HR, hazard ratio; CI, confidence interval; β2M, β_2_-microglobulin; CrCl, creatinine clearance; ASCT, autologous stem cell transplantation; ECOG, Eastern Cooperative Oncology Group; LDH, lactate dehydrogenase*
