# Supplementary material for: Prognostic Significance of Isolated Beta 2-Microglobulin Elevation in Thai Multiple Myeloma: Impact of Renal Function Assessment
Source: J Hematol. 2026 Jun 20;15(3):144–55. doi: 10.14740/jh2212 (PMC13375423; doi:10.14740/jh2212)
Supplement: Suppl 6 — Stratified multivariable Cox proportional hazards analysis for overall survival (OS). The model was stratified by era of diagnosis (2006–2010, 2011–2015, 2016–2020, and 2021–2023) to account for potential variation in baseline hazards across treatment eras. [file jh-15-03-144-s006.docx]

**Suppl 6.** Stratified multivariable Cox proportional hazards analysis for overall survival (OS). The model was stratified by era of diagnosis (2006–2010, 2011–2015, 2016–2020, and 2021–2023) to account for potential variation in baseline hazards across treatment eras.

| **Variable** | **Adjusted HR (95% CI)** | ***P* value** |
| --- | --- | --- |
| **Study groups (CrCl-based)** |  |  |
| Group A (Low β2M) | 1.00 (Reference) | — |
| Group B (High β2M, CrCl ≥60 mL/min) | 2.67 (1.17–6.07) | 0.019 |
| Group C (High β2M, CrCl <60 mL/min) | 1.74 (1.08–2.79) | 0.022 |
| **Patient characteristics** |  |  |
| Age (per year increase) | 1.00 (0.98–1.02) | 0.992 |
| Sex (Male vs. Female) | 1.85 (1.14–3.01) | 0.013 |
| ECOG performance status (≥2 vs. 0–1) | 2.21 (1.34–3.65) | 0.002 |
| **Disease & treatment** |  |  |
| Plasmacytoma (Yes vs. No) | 1.29 (0.82–2.04) | 0.268 |
| Bortezomib induction (Yes vs. No) | 0.73 (0.46–1.14) | 0.167 |
| Autologous SCT (Yes vs. No) | 0.38 (0.20–0.73) | 0.004 |

*Abbreviations: HR, hazard ratio; CI, confidence interval; β2M, β₂-microglobulin; CrCl, creatinine clearance; ECOG, Eastern Cooperative Oncology Group; SCT, stem cell transplantation.*
